# Supplementary material for: DNA Methylation Profiling for Diagnosing Undifferentiated Sarcoma with Capicua Transcriptional Receptor (CIC) Alterations
Source: Int J Mol Sci. 2020 Mar 6;21(5):1818. doi: 10.3390/ijms21051818 (PMC7084764; doi:10.3390/ijms21051818)
Supplement: Supplementary file 1 [file ijms-21-01818-s001.pdf]

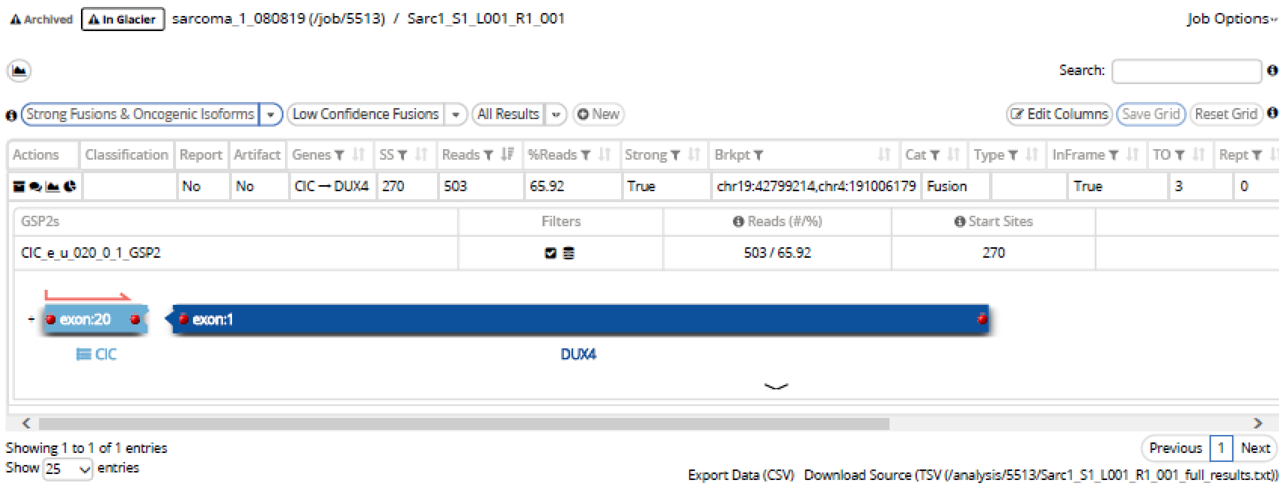

Supplementary Figure 1: Archer analysis software version 5.0 reveals the CIC-DUX4 rearrangement: CIC (Exon20)→DUX4 (Exon1) (chr19:42799214,chr4:191006179) detected in our patient.
